# Perception of Face and Body Expressions Using Electromyography, Pupillometry and Gaze Measures

**DOI:** 10.3389/fpsyg.2013.00028

**Published:** 2013-02-08

**Authors:** Mariska E. Kret, Jeroen J. Stekelenburg, Karin Roelofs, Beatrice de Gelder

**Affiliations:** ^1^Psychology Department, University of AmsterdamAmsterdam, Netherlands; ^2^Cognitive and Affective Neurosciences Laboratory, Tilburg UniversityTilburg, Netherlands; ^3^Radboud University Nijmegen, Behavioural Science Institute and Donders Institute for Brain Cognition and BehaviourNijmegen, Netherlands; ^4^Psychology and Neuroscience Department, Maastricht UniversityMaastricht, Netherlands

**Keywords:** facial expressions, emotional body language, scenes, pupil dilation, fixations, electromyography

## Abstract

Traditional emotion theories stress the importance of the face in the expression of emotions but bodily expressions are becoming increasingly important as well. In these experiments we tested the hypothesis that similar physiological responses can be evoked by observing emotional face and body signals and that the reaction to angry signals is amplified in anxious individuals. We designed three experiments in which participants categorized emotional expressions from isolated facial and bodily expressions and emotionally congruent and incongruent face-body compounds. Participants’ fixations were measured and their pupil size recorded with eye-tracking equipment and their facial reactions measured with electromyography. The results support our prediction that the recognition of a facial expression is improved in the context of a matching posture and importantly, vice versa as well. From their facial expressions, it appeared that observers acted with signs of negative emotionality (increased corrugator activity) to angry and fearful facial expressions and with positive emotionality (increased zygomaticus) to happy facial expressions. What we predicted and found, was that angry and fearful cues from the face or the body, attracted more attention than happy cues. We further observed that responses evoked by angry cues were amplified in individuals with high anxiety scores. In sum, we show that people process bodily expressions of emotion in a similar fashion as facial expressions and that the congruency between the emotional signals from the face and body facilitates the recognition of the emotion.

## Introduction

The communication of emotion includes recognizing signals of hostility or joy and reacting to signals of distress. Humans are especially sensitive to facial expressions and gestural signals of others, and use these signs to guide their own behavior. Previous research has largely focused on the perception of facial expressions (Haxby et al., 2000; Adolphs, 2002). But our ability to communicate also relies heavily on decoding messages provided by body postures (de Gelder et al., 2004, 2010; de Gelder, 2006; Kret et al., 2011c). The first goal of the current study is to test to what extent facial expressions are recognized and processed as a function of the accompanied body posture and vice versa. Second, research has shown that highly anxious individuals respond stronger to facial expressions than those with a low anxiety level (MacLeod and Cohen, 1993; Amin et al., 1998; Miers et al., 2008). Our second goal is to test whether highly anxious people are also hyper-reactive to body postures.

Before we lay out our research questions, we start with an overview on how humans generally recognize and react to emotional expressions and describe similarities and differences between faces and bodies in terms of the mechanisms involved in emotion expression and perception. Finally, we describe individual differences in these mechanisms with a focus on anxiety.

The perception of bodily expressions is a relatively novel topic in affective neuroscience, a field dominated so far by investigations of facial expressions. But faces and bodies are equally salient and familiar in daily life and often convey the same information about identity, emotion, and gender. Moreover, emotions from both sources are usually very well recognized as shown in different validation studies. The recognition rate of angry, fearful, and happy emotions is especially high (for the NimStim facial expression set, these emotions were correctly recognized at 74.3% with nine response alternatives, and the body postures in the Bodily Expressive Action Stimulus Test (BEAST) set were correctly recognized at 92.5% with four response alternatives). Even when these stimuli are presented subliminally, recognition tends to be well above chance (Esteves et al., 1994; Dimberg et al., 2000; Stienen and de Gelder, 2011a,b).

In addition to facial expressions, bodily expressions give us information about the action tendency of the agent. Aggressive body postures therefore, can be perceived as a more direct threat to physical harm than facial expressions (de Gelder et al., 2010). When we observe another individual, such as a friend expressing his or her anger toward us, different processes are initiated. First, the *attention* is drawn toward the threat, especially toward our friends’ face or eyes (Green et al., 2003; Lundqvist and Öhman, 2005; Fox and Damjanovic, 2006) and toward his body posture (Bannerman et al., 2009). Next, *we become aroused too*: our heart beat changes, we start sweating, and our pupils dilate (Bradley et al., 2008; Gilzenrat et al., 2010). Moreover, it is possible that the observed emotion will be reflected in our own facial expression (Dimberg, 1982).

The perception of facial expressions and body postures is interactive and context-dependent (faces: Righart and de Gelder, 2006; Kret and de Gelder, 2012a; bodies: Kret and de Gelder, 2010). Meeren et al. (2005) show that observers judging a facial expression are strongly influenced by emotional body language; an amplitude increase of the occipital P1 component 115 ms after stimulus presentation onset points to the existence of a rapid neural mechanism sensitive to the agreement between simultaneously presented facial and bodily emotional expressions. Continuing this line of research, Aviezer et al. (2008) positioned prototypical pictures of disgusted faces on torsos conveying different emotions. Their results showed that combining a facial expression and a torso but sometimes also showing an object (for example underwear) induced changes in the recognition of emotional categories from the facial expressions to the extent where the “original” basic expression was lost when positioned on an emotionally incongruent torso.

Research has shown that whereas the immediate expression of emotions by the face and the body is automatic and predominantly regulated by subcortical structures (Aggleton, 2000; Lanteaume et al., 2007), the conscious regulation of emotional expressions (smiling during a job interview or hiding joy in a poker play) is steered by higher order cortical structures such as the orbitofrontal cortex (Damasio, 1994). Some people, such as those with an anxious personality type become socially inhibited because, in social interactions, they over-activate this network, which takes so much cognitive effort that it has a negative effect on the interaction (Kret et al., 2011a). Anxious individuals have a propensity for over-responding to social or emotional signals and in particular to those that are threatening. This hyper-responsiveness may translate to increased activation in the amygdala (Etkin et al., 2004; Hayes et al., 2012), increased attention toward threat (Bar-Haim et al., 2007) paired with increased pupil dilation (Kimble et al., 2010) and altered facial expressions as measured with electromyography (EMG; Dimberg and Thunberg, 2007). In addition, when confronted with facial expressions, they may attend to the wrong cues (Horley et al., 2003; Bar-Haim et al., 2005; Mogg et al., 2007). Moreover, highly anxious subjects are likely to give negative interpretations of ambiguous social situations in which conflicting information is presented (Huppert et al., 2007). Previous studies have suggested that anxious individuals prefer negative interpretations over other possibilities when facial expressions convey conflicting information (e.g., Richards et al., 2002; Yoon and Zinbarg, 2008). Most studies so far used facial expressions. But a recent study looked at vocalizations as well (Koizumi et al., 2011). They showed that anxious individuals when recognizing emotions from either the face or the voice in paired combinations were more likely to interpret others’ emotions in a negative manner, putting more weight on the to-be-ignored angry cues. This interpretation bias was found regardless of the cue modality (i.e., face or voice). Interestingly, anxiety did not affect recognition of the face or voice cues when presented in isolation. Therefore, this interpretation bias is due to poor integration of the face with simultaneously presented other cues such as voice cues among anxious individuals. We now would like to test whether anxious individuals also hyper-react to negative emotions expressed by the body and whether they would misinterpret positive emotions when conflicting cues from the face or the body are presented simultaneously.

### Objectives of the current study

We investigated the recognition of emotions from the face and the body separately, and when combined with a matching or non-matching whole body. In *Experiment 1*, participants categorized happy, angry, and fearful isolated faces and happy, angry, and fearful isolated bodies. In *Experiment 2*, the same participants were asked to categorize emotions in facial expressions, but the face presented was on top of a body that expressed either the same, or a different emotion. *Experiment 3* used the same stimuli as Experiment 2, but participants were now asked to label the body emotion and ignore the facial expression. The experiments were given in a random order. We tested three main hypotheses:

1) We predicted that recognition of facial and bodily expressions would be improved when shown paired with an emotionally congruent face or body.
2) Regarding the overall fixation patterns, we expected that angry and fearful cues would attract more attention than happy cues.
3) We predicted that anxious participants would respond stronger to angry and fearful cues from the face and the body (longer fixation durations, greater pupillary response, and enhanced corrugator activity) than to happy cues. Moreover, we predicted that they would recognize happy cues more often as negative signals when these happy cues were combined with an angry or fearful context.

#### Experiment 1. Categorizing isolated facial and bodily expressions of emotion

## Materials and Methods

### Participants

Thirty-seven students from Tilburg University (26 females, mean age 22.7, range 19–29 years old; 11 males; mean age: 23.8, range 19–32 years old) provided informed consent and took part in the experiment. All participants were included in the analyses except in the EMG analyses due to technical problems with the EMG data of four participants in Experiment 1 and three in Experiment 2 which were not recorded. The other data from these participants could be analyzed so they were not excluded from any other analyses. For Experiment 3, data for all participants was properly recorded and included in the analyses. Participants had no neurological or psychiatric history, were right-handed and had normal or corrected-to-normal vision. The study was performed in accordance with the Declaration of Helsinki and approved by the local medical ethical committee.

### Materials

**Figure 1:**
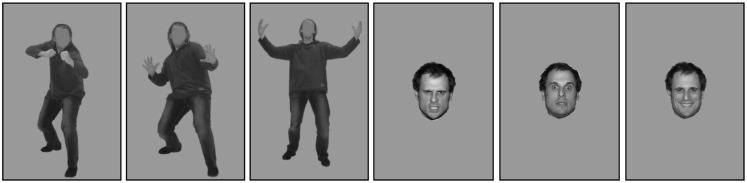
**Stimulus examples**. Bodily (blurred facial features) and facial expressions of emotion.

Fearful, happy, and angry facial expressions of six male individuals that were correctly recognized above 80% were selected from the NimStim set (Tottenham et al., 2009). The corresponding bodily expressions were taken from the BEAST stimulus database (de Gelder and Van den Stock, 2011). For the current study, we selected the best models, with recognition scores above 80% correct. We used only male bodies because we previously found that these evoke stronger arousal when anger and fear are expressed (Kret et al., 2011b; Kret and de Gelder 2012b). Pictures were presented in grayscale, against a gray background. Using Photoshop the luminance of each stimulus was modified to the average luminance. A final check was made with a light meter on the test computer screen. The size of the stimuli was 354 × 532 pixels (see Figure 1).

### Procedure

After attaching the electrodes to the participants’ face, the eye-tracking device was positioned on the participant’s head. Next, a nine-point calibration was performed and repeated before each block. Stimuli were presented using E-prime software on a PC screen with a resolution of 1024 by 768 and a refresh rate of 100 Hz. Each trial started with a fixation cross, shown for minimally 3000 ms until the participant fixated and a manual drift correction was performed by the experiment leader, followed by a picture presented for 4000 ms and a gray screen (3000 ms). The face and body stimuli were randomly presented within two separate blocks containing 36 trials each. To keep participants naive regarding the purpose of the EMG, they were told that the electrodes recorded perspiration. The order of the two blocks and also the order of the experiments were counterbalanced. Two additional passive viewing tasks had been given (results will be published elsewere). Participants were asked to categorize the emotion being depicted, choosing amongst three response alternatives that were written on the screen (anger, fear, happy) and three corresponding buttons on a button-box. The order of the emotion labels was counterbalanced. Participants were requested to indicate their choice after the stimulus disappeared from the screen.

### Measurements

#### Facial EMG

The parameters for facial EMG acquisition and analysis were selected according to the guidelines by van Boxtel (2010). BioSemi flat-type active electrodes were used and facial EMG was measured bipolarly over the zygomaticus major and the corrugator supercilii on the right side of the face at a sample rate of 1024 Hz. The common mode sense (CMS) active electrode and the driven right leg (DRL) passive electrode were attached to the left cheek and used as reference and ground electrodes, respectively (http://www.biosemi/faq/cmsanddrl.htm). Before attachment, the skin was cleaned with alcohol and the electrodes were filled with electrode paste. Raw data were first filtered offline with a 20–500 Hz band-pass in Brain Vision Analyzer Version 1.05 (Brain Products GmbH), and full-wave rectified. Data were visually inspected for excessive movement during baseline by two independent raters who were blind to the trial conditions. Trials that deemed problematic were discarded, resulting in the exclusion of 6.07% (SD 7.50) of the trials from subsequent analysis. Due to technical problems, the EMG data of four participants in Experiment 1 and three in Experiment 2 were not recorded. Subsequently, mean rectified EMG was calculated across a 4000-ms post-stimulus epoch, and a 1000 ms pre-stimulus baseline period. Mean rectified EMG was expressed as a percentage of the mean pre-stimulus baseline EMG amplitude. Percentage EMG amplitude scores were averaged across valid trials and across emotions.

The zygomaticus is predominantly involved in expressing happiness. The corrugator muscle can be used to measure the expression of negative emotions including anger and fear (van Boxtel, 2010). In order to differentiate between these two negative emotions, measuring additional face muscles such as the frontalis would be necessary (Ekman and Friesen, 1978). However, this was not possible in the current experiment, due to the head-mounted eye-tracker. Activity of the corrugator in a specific context, such as by presenting clear emotional stimuli, can be interpreted as the expression of the observed emotion (Overbeek et al., 2012).

#### Eye-tracking

Eye movements were recorded with a sample rate of 250 Hz using the head-mounted EyeLink Eye-Tracking System (SensoMotoric Instruments GmbH, Germany). A drift correction was performed on every trial to ensure that eye gaze data were adjusted for movement. We used the default Eyelink settings which defined a blink as a period of saccade detector activity with the pupil data missing for three or more samples in a sequence. A saccade was defined as a period of time where the saccade detector was active for two or more samples in sequence and continued until the start of a period of saccade detector inactivity for 20 ms. The configurable acceleration (8000 degrees/s) and velocity (30 degrees/s) threshold were set to detect saccades of at least 0.5° of visual angle. A fixation was defined as any period that was not a blink or saccade. Analyses were performed on the proportion of time spent looking at each interest area within the time spent looking on the screen, with the first 200 ms discarded due to the fixed position of the fixation cross. In accordance with previous literature, blinks were linearly interpolated before subtracting a 500 ms baseline from the average pupil size during the last 2 s of picture presentation. The first 2 s were not included in the analysis to avoid influences of the initial dip in pupil size (Bradley et al., 2008).

#### Anxiety measure

On the day before testing, participants filled out the STAI Trait Measure (Spielberger, 1983). The average score was within the normal range 49.89 (standard deviation: 1.75, range: 46–54). The reason for giving this questionnaire on the day beforehand rather than after the experiment was to avoid possible influences of the task.

### Data analysis

Data from the different measurements were analyzed in separate ANOVAs with two body parts: (head and body) and three emotions (anger, fear, happiness). Due to technical failure, the EMG data of four participants were not recorded. Significant main effects were followed up by Bonferroni-corrected pairwise comparisons and interactions with two-tailed *t*-tests. In separate multiple linear regression models, we investigated the influence of anxiety, as measured with the STAI.

## Results

Participants categorized isolated facial and bodily expressions of anger, happiness and fear while their fixation patterns, pupil dilation, and facial muscle movements were being recorded. The objective of this experiment was to investigate whether isolated emotional expressions from the face and the body are processed similarly.

### Accuracy

There were main effects of body part and emotion [*F*(1, 36) = 87.00, *p* < 0.001; *F*(2, 72) = 12.64, *p* < 0.001] and an interaction between emotion and body part [*F*(2, 72) = 15.092, *p* < 0.001]. Faces were recognized at ceiling, and better than bodies (face: Mean = 0.985, SE = 0.004, body: Mean = 0.865, SE = 0.013) and as such there was no significant difference between the three *facial* expressions (although happy faces were slightly better recognized than fearful ones, Mean = 0.991, SE = 0.004 versus Mean = 0.973, SE = 0.010), but pairwise comparisons of the body postures showed that angry and fearful bodies were better recognized than happy ones (anger: Mean = 0.944, standard error (SE) = 0.015; happy: Mean = 0.757, SE = 0.037; fear: Mean = 0.896, SE = 0.015; *p*s < 0.01). The multiple linear regression model that included the accuracy rates per condition was significant [*F*(6, 28) = 2.64, *p* < 0.05]. A positive relation was found between the STAI and the recognition of fearful faces (β = 0.382, *t* = 2.217, *p* < 0.05) and a negative relation with the recognition of fearful bodies (β = 0.386, *t* = 2.424, *p* < 0.05) (see Figure 3).

### Gaze and fixation behavior

**Figure 2:**
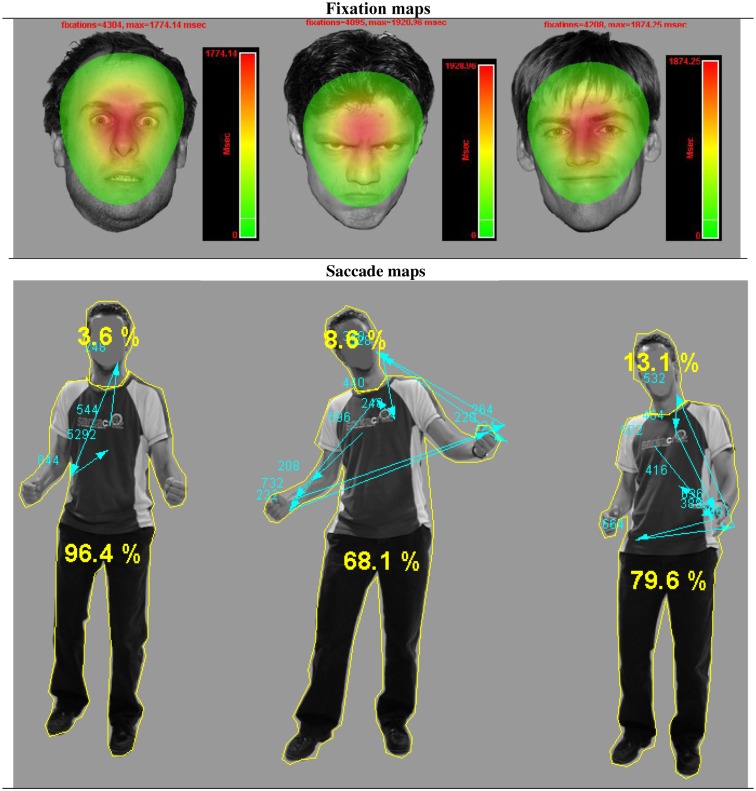
**Gaze fixations on emotional face and body expressions**. Fixation and saccade maps of one participant on three different trials. The fixation duration based heat maps show data from all participants on all trials per emotional face condition. For visualization purposes, these heat maps are presented against a background of one exemplar stimulus from that condition. The heat maps show that participants had more fixations on fearful faces, covering a greater area, yet with a clear center on the eye region. The picture with the body postures shows the distribution of fixations and saccades of one participant on three different trials. The yellow lines around the head and body are the interest areas and the yellow numbers, the percentage of the total fixation duration that fell in either the face or the body ROI. The blue arrows indicate the saccades, starting from the chest (fixation cross) and the blue numbers the fixation durations.

There was a main effect of body part *F*(1, 36) = 304.06, *p* < 0.001 and of emotion *F*(2, 72) = 184.81, *p* < 0.001. Participants looked (as a proportion of the whole screen) longer at faces than at bodies (Mean = 0.998, SE = 0.003 versus *M* = 0.553, SE = 0.025, *p*s < 0.001) and at angry and fearful more than at happy expressions (anger: Mean = 0.814, SE = 0.014 and fear: Mean = 0.806, SE = 0.014 versus happy Mean = 0.691, SE = 0.013). There was no difference between anger and fear (*p* = 0.652). However, there was an interaction between body part and emotion *F*(2, 72) = 186.37, *p* < 0.001 that showed that these effects were fully driven by the body. This was confirmed with an ANOVA that included only body postures. Happy postures were less attended to than either angry or fearful postures *F*(2, 72) = 207.26, *p* < 0.001 (Mean = 0.396, SE = 0.025 versus Mean = 0.637, SE = 0.027 and Mean = 0.625, SE = 0.027, *p*s < 0.001). There was no effect of emotion on fixation duration on the whole face (*p* = 0.380). However, we found an effect of emotion on the duration of fixations on the eyes *F*(2, 72) = 64.32, *p* < 0.001. Participants attended longest to fearful eyes (Mean = 0.314, SE = 0.017 versus anger: Mean = 0.144, SE = 0.011 and happy: 0.234, SE = 0.017, *p*s < 0.001) (see Figure 2).

### Electromyography

There was an interaction between emotion and body part on the zygomaticus *F*(2, 68) = 6.15, *p* < 0.005. When analyzing the zygomaticus response to the different emotions separately for faces and for bodies, it appeared that this facial muscle only differentially responded to facial expressions *F*(2, 68) = 4.35, *p* < 0.05 and was more active following happy than angry faces (Mean = 115.480, SE = 4.994 versus Mean = 103.830, SE = 3.074, *p* < 0.05) (fear: Mean = 106.835, SE = 3.144). The corrugator showed a main effect of body part *F*(1, 34) = 17.35, *p* < 0.001 and was more responsive to bodies than faces (Mean = 105.033, SE = 0.952 versus Mean = 99.656, SE = 0.762). There was another main effect for emotion *F*(2, 68) = 7.31, *p* < 0.001, showing a greater response following fearful (and to some extent angry) than happy expressions (Mean fear: 103.749, SE = 0.802 and Mean anger: 102.406, SE = 0.583 versus Mean happy: 100.879, SE = 0.749, *p* < 0.005; *p* = 0.081). Anger and fear did not differ (*p* = 0.287). The marginally significant interaction between body part and emotion *F*(2, 68) = 2.62, *p* = 0.080 however, suggests that the main effect of emotion is driven by the facial expression. Analyzing the response to faces only showed again a main effect of emotion *F*(2, 68) = 13.62, *p* < 0.001, with greater responses for angry and fearful versus happy faces (Mean = 100.354, SE = 0.836 and Mean = 101.525, SE = 0.880, Mean = 97.089, SE = 1.010, *p-*values < 0.001). There was no emotion effect for bodies (*p* = 0.472). The multiple linear regression model that included all EMG responses (corrugator and zygomaticus) per condition was highly significant *F*(12, 22) = 5.092, *p* = 0.0005. There was a positive relation between the STAI and zygomaticus response following angry faces (β = 0.399, *t* = 2.738, *p* < 0.05). However, this “smile,” was also paired with a frown, as there was a marginally significant relation between the STAI and corrugator activity following angry and happy faces (β = 0.262, *t* = 1.514, *p* = 0.1; β = 0.319, *t* = 1.933, *p* = 0.07). There was a positive relationship between the STAI and corrugator activity following angry bodies (β = 0.380, *t* = 2.841, *p* < 0.01). However, there were negative relationships between the STAI and zygomaticus activity following fearful and happy bodies (β = 0.352, *t* = 2.404, *p* < 0.05; β = 0.451, *t* = 2.727, *p* < 0.05).

### Pupil size

There was a main effect of body part *F*(1, 36) = 18.64, *p* < 0.001. Pairwise comparisons revealed greater pupil dilation following bodies than faces (Mean = 173.320, SE = 16.048 versus Mean = 94.530, SE = 18.380, *p* < 0.001), probably due to the differences in size of the image (see Figure 1). In both cases, for faces and for bodies, the magnitude of the response was consistent with expectations (anger and fear > happy) but not significantly. For comparable results see Bradley et al. (2008). The multiple linear regression model that included the pupil sizes per condition was marginally significant *F*(6, 29) = 2.305, *p* = 0.06. There was a positive relation between the STAI and pupil size following angry faces (β = 0.587, *t* = 2.488, *p* < 0.05).

### Discussion experiment 1

We used facial EMG, pupillometry, and gaze to measure similarities in the processing of body postures and facial expressions. Angry and fearful body postures and fearful eyes were the most frequent gaze targets. Participants reacted to the sight of the facial expressions with the expected muscular activity but not to body expressions as was previously reported (Magnée et al., 2007; Tamietto et al., 2009). But in line with the study by Magnée et al. (2007), we found that the corrugator responded more to bodies than to faces. One difference between the current and the previous studies is the addition of angry expressions. Adding this third emotion made the task more difficult which may be a reason for the larger differences between individuals in the current study. Moreover, the study by Tamietto et al. (2009) included only two participants with visual cortex blindness. A third difference is that in the current study we used only male actors. These task differences may explain the lack of differentiation of EMG signals between observing different bodily expressions.

With regard to anxiety state, we indeed observed hyper-reactivity to emotional cues (MacLeod and Cohen, 1993; Amin et al., 1998; Miers et al., 2008). Anxious individuals showed a greater corrugator response to angry body postures and to angry faces (for similar results, see Dimberg and Thunberg, 2007). But in the latter case, this frown was paired with a smile. The meaning of the smile could be a sign of submission, a conciliatory smile which was paired with high arousal, as shown by their greater pupil dilation. A similar finding has been reported previously in subjects with a dismissing-avoidant pattern of attachment (characterized by repressing anxiety-related signals) who showed an increased zygomaticus response (“smiling reaction”) to angry faces (Sonnby-Borgström and Jönsson, 2004). In addition, we found that the more anxious subjects were, the better they were in decoding fearful faces, but the more difficulties they had in recognizing this emotion from body cues.

In the next experiments, we combine facial and bodily expressions in a face and a body categorization task. The goal is to test the influence of body expressions on the recognition of and responses to facial expressions and vice versa. In addition, the role of anxiety is investigated.

#### Experiment 2. Categorizing facial expressions of emotion in the context of body expressions

In this experiment, participants (see Participants, Exp. 1 for details) categorized facial expressions that were presented together with emotionally congruent or incongruent body postures. The purpose of this study is to investigate whether recognition is facilitated with the presence of a congruent body posture and, in addition, whether the body expression influences not only how the face is perceived, but also how it is processed.

##### Procedure

Materials consisted of the same face and body images used in Experiment 1 but here the faces and bodies were combined in emotionally congruent and incongruent pairs (see Figure 4). The identity-pairs were kept the same across the three emotions, making nine combinations. The stimuli were divided in two blocks containing 36 random trials each with 18 congruent and 18 incongruent stimuli (72 trials in total). Participants were requested to label the facial expression. Thus, in order to perform well on this task, participants had to look at the face and ignore the bodily expression. On average, they spend 59% of their looking time at the face and 9% at the body. After the experiment, they were asked to describe what they had seen. All participants mentioned having seen emotional expressions. Most of them noticed that in some cases the facial and bodily expressions were incongruent.

##### Data analysis

Data from the different measurements were analyzed in separate ANOVAs with three facial expressions × three bodily expressions (anger, fear, happiness). To analyze the eye-tracking data, we created two regions of interest (ROIs): the face and the body. Due to a technical failure, the EMG data of three participants were not recorded. Significant main effects were followed up by Bonferroni-corrected pairwise comparisons and interactions with two-tailed *t*-tests.

##### Results

**Figure 3:**
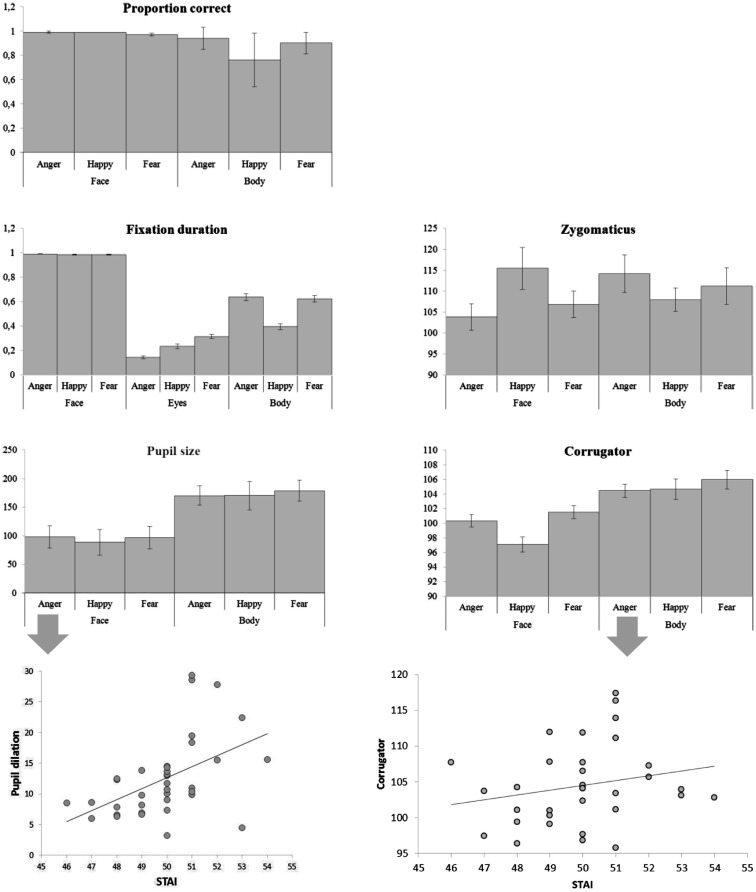
**Categorizing facial and bodily expressions of emotion**. Means of all measurements. The error bars represent the standard error.

*Accuracy*. There were main effects for facial expression *F*(2, 72) = 17.64, *p* < 0.001 and body expression *F*(2, 72) = 3.37, *p* < 0.05 and an interaction between face and body expression *F*(4, 144) = 9.75, *p* < 0.001. Pairwise comparisons revealed no differences between the body postures. Happy faces were better recognized than angry or fearful faces (happy: Mean = 0.984, SE = 0.007, anger: Mean = 0.968, SE = 0.010, fear: Mean = 0.887, SE = 0.020). In line with previous literature, participants were better in recognizing angry and fearful faces when accompanied with emotionally congruent versus incongruent bodies (angry face congruent versus angry face incongruent: Mean = 0.993, SE = 0.005 versus Mean = 0.956, SE = 0.016; fearful face congruent versus fearful face incongruent: Mean = 0.946, SE = 0.017 versus Mean = 0.857, SE = 0.027) *t*(36) ≥ 2.79,*p* < 0.01 (happy faces were recognized at ceiling; Meeren et al., 2005) (see Figure 3). Relations between recognition rates for the different conditions and the STAI score were investigated in a multiple regression model but this model was not significant (*p* > 0.05).

**Figure 4:**
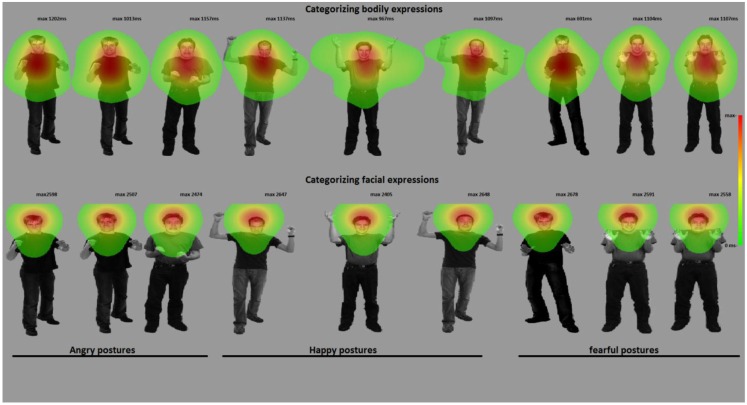
**Categorizing facial and bodily expressions of emotion**. The figure shows one stimulus exemplar per condition with a superimposed fixation map (duration based and averaged per condition). In experiment 2, participants categorized facial expressions, whereas in experiment 3, they categorized bodily expressions. For visualization purposes, these heat maps are presented against a background of one exemplar stimulus from that condition.

*Gaze and fixation behavior*. There was a main effect of facial expression on fixation duration on the face ROI *F*(2, 72) = 22.21, *p* = 0.001. A face was looked at longest when it expressed anger (anger: Mean = 0.657, SE = 0.034, happy: Mean = 0.552, SE = 031, fear: Mean = 0.551, SE = 0.033, *p*-values < 0.001). Body posture did not affect fixation durations on the face ROI (*p* = 0.426) (see Figures 3 and 4).

*Electromyography*. The zygomaticus reacted to the expression that was shown by the face, independent of the bodily expression *F*(2, 68) = 4.67, *p* = 0.012. Increased responses of happy versus angry (Mean = 111.948, SE = 2.946 versus Mean = 105.681, SE = 2.099, *p* < 0.01) and fearful faces (Mean = 105.860, SE = 3.358, *p* < 0.05) were observed. The corrugator also responded to the observed face *F*(2, 68) = 5.29, *p* < 0.01 and was more active for fearful versus happy expressions (Mean = 103.118, SE = 1.006 versus Mean = 100.169, SE = 0.739, *p* < 0.05; numerically consistent for angry (Mean = 102.730, SE = 1.161) versus happy faces *p* = 0.120). Relations between EMG responses to the different stimulus conditions and the STAI score were investigated in a multiple regression model but this model was not significant (*p* > 0.05) (see Figure 3).

*Pupil size*. Participant’s pupils responded to all emotional expressions, as compared to baseline (*p*-values < 0.005) but there was no difference between the emotions (see Table 1 for all means). Relations between pupil size responses to the different stimulus conditions and the STAI score were investigated via multiple regression. This model was significant (*F*(9, 26) = 2.454, *p* < 0.05). There was a positive relationship between the STAI and pupil size in the condition where fearful faces were paired with happy bodies (β = 0.646, *t* = 2.156, *p* < 0.05) (see Figure 3).

**Table 1:** **Means and standard errors**.

Expression		Fixation duration on face		Zygomaticus		Corrugator		Pupil size		Face recognition	
Body	Face	Mean	SE	Mean	SE	Mean	SE	Mean	SE	Mean	SE
Anger	Anger	0.66	0.03	109.67	3.91	102.93	1.37	89.12	18.37	0.99	0.01
	Happy	0.56	0.04	112.90	4.35	100.88	1.32	65.83	19.92	0.99	0.01
	Fear	0.53	0.04	107.48	3.88	102.32	1.21	103.51	27.12	0.87	0.02
Happy	Anger	0.65	0.03	106.01	2.70	102.57	1.48	134.65	32.40	0.96	0.01
	Happy	0.52	0.03	110.75	3.27	99.91	0.90	90.78	20.98	0.99	0.01
	Fear	0.57	0.04	102.04	2.16	103.92	1.47	93.12	26.08	0.85	0.03
Fear	Anger	0.66	0.04	101.37	2.41	102.68	1.30	96.28	25.39	0.95	0.02
	Happy	0.58	0.04	112.19	4.37	99.72	0.73	63.64	24.51	0.97	0.01
	Fear	0.56	0.03	108.06	5.43	103.11	0.98	106.96	25.08	0.95	0.02

### Discussion experiment 2

In this experiment, we investigated how participants perceive and categorize a facial expression presented in the context of a bodily expression. As expected, recognition of facial expressions improved when the body and face showed the same expression. We did not find an overall hyper-responsiveness in highly anxious subjects but we observed a specific increase in arousal (as measured by greater pupil dilation) in the condition where fearful faces were paired with happy bodies. In the next experiment, participants are asked to categorize the body posture and ignore the face.

#### Experiment 3. Categorizing bodily expressions of emotion in the context of facial expressions

In this experiment, the exact same stimuli were shown as in the previous experiment, but under different task instructions. Participants (see Participants, Exp. 1 for details) here were asked to attend to and categorize the body posture and ignore the facial expression. On average, they spend 58% looking at the body and 23% at the face.

##### Data analysis

Data from the different measurements were analyzed in separate ANOVAs with three facial expressions × three bodily expressions (anger, fear, happiness). Significant main effects were followed up by Bonferroni-corrected pairwise comparisons and interactions with two-tailed *t*-tests.

##### Results

*Accuracy*. There were two main effects and an interaction [face: *F*(2, 72) = 4.91, *p* < 0.01; body: *F*(2, 72) = 24.15, *p* < 0.001; face × body: *F*(4, 144) = 4.88, *p* < 0.005]. Accuracy was lowest for happy bodies (happy: Mean = 0.749, SE = 0.033 versus anger: Mean = 0.953, SE = 0.018 and fear: Mean = 0.905, SE = 0.014, *p*-values < 0.001), providing most room for an influence of facial expressions. Fear and anger were not significantly different (*p* = 0.104). As expected, happy bodies were recognized better in combination with a happy versus fearful or angry face (happy body congruent versus happy body incongruent: Mean = 0.914, SE = 0.014 versus Mean = 0.901, SE = 0.018) *t*(36) ≥ 2.73, both *p*-values < 0.01. The multiple regression model was not significant *F*(9, 26) = 1.485, *p* = 0.20. We predicted that anxious individuals would make mistakes when categorizing a happy posture in the context of an angry face. Indeed, the recognition rates in this condition were the only significant predictor in this model β = 0.844, *t* = 2.551, *p* = 0.01.

*Gaze and fixation behavior*. There was a main effect for body posture *F*(2, 72) = 124.82, *p* < 0.001. Participants attended longer to the body in the case of a threatening posture (anger: Mean = 0.649. SE = 0.022, fear: Mean = 0.642, SE = 0.020, happy: Mean = 0.463, SE = 0.020, *p*s < 0.001). There was also a main effect for facial expression *F*(2, 72) = 6.41, *p* < 0.005. Participants attended longer to the body when the face expressed happiness versus fear (Mean = 0.603, SE = 0.021 versus Mean = 0.566, SE = 0.021, *p* < 0.01, which was numerically consistent for anger, Mean = 0.584, SE = 0.019). The interaction was not significant *F*(4, 144) = 2.04, *p* = 0.093. Because participants still spent about a quarter of their time observing the face, we were able to analyze the effect of facial and bodily expressions on the looking times within the face ROI. There were main effects for facial expression and bodily expression on fixation durations within the face ROI *F*(2, 72) = 3.69, *p* < 0.05; *F*(2, 72) = 9.00, *p* < 0.001. Participants attended longer to fearful than angry (Mean = 0.243, SE = 0.02 versus Mean = 0.215, SE = 0.018, *p* < 0.05) or happy faces (Mean = 0.223, SE = 0.018, *p* = 0.161, ns). Interestingly, the looking times on the face depended mostly on the bodily expression, being longest when the body posture expressed happiness versus fear (Mean = 0.254, SE = 0.017 versus Mean = 0.207, SE = 0.019, *p* < 0.001) or anger (Mean = 0.219, SE = 0.021, *p* < 0.05).

*Electromyography*. There was a trend toward a main effect for body expression on the zygomaticus *F*(2, 66) = 2.73, *p* = 0.073 but follow-up pairwise comparisons did not yield any significant difference (happy versus angry bodies; Mean = 109.916, SE = 3.596 versus Mean = 102.785, SE = 2.130, *p* = 0.115). The corrugator did not show an effect of facial or bodily expression. The multiple regression model was significant *F*(18, 15) = 3.625, *p* < 0.01. We found a positive relation between the STAI and EMG activity of both the zygomaticus and the corrugator in the condition where angry faces were paired with fearful bodies (β = 0.614, *t* = 3.162, *p* < 0.01; β = 1.287, *t* = 2.488, *p* < 0.05). A positive relation was also found with the zygomaticus in the condition where happy faces were paired with angry bodies (β = 0.656, *t* = 3.152, *p* < 0.01).

*Pupil size*. Pupil dilation showed an increase in activity as compared to baseline *t*(36) ≥ 7.035, all *p*-values < 0.001 but did not respond more to one emotion than the other. The multiple regression model was not significant *p* > 0.05 (see Table 2 for all means and SEs).

**Table 2:** **Means and standard errors**.

Expression		Fixation duration on face		Fixation duration on body		Zygomaticus		Corrugator		Pupil size		Body recognition	
Body	Face	Mean	SE	Mean	SE	Mean	SE	Mean	SE	Mean	SE	Mean	SE
Anger	Anger	0.20	0.02	0.67	0.02	103.30	2.87	101.95	0.58	160.07	14.46	0.95	0.02
	Happy	0.22	0.02	0.67	0.02	104.68	3.40	100.94	1.22	119.48	13.24	0.95	0.02
	Fear	0.24	0.03	0.61	0.03	100.37	3.33	101.82	0.91	135.42	18.51	0.97	0.02
Happy	Anger	0.24	0.02	0.46	0.02	109.06	4.84	101.95	1.01	131.27	15.17	0.72	0.04
	Happy	0.24	0.02	0.48	0.02	109.22	6.34	101.11	1.05	129.56	17.61	0.81	0.03
	Fear	0.28	0.02	0.44	0.02	111.47	6.06	102.07	1.25	123.27	17.46	0.72	0.04
Fear	Anger	0.20	0.02	0.62	0.02	102.60	2.90	102.93	1.45	144.54	20.55	0.89	0.02
	Happy	0.21	0.02	0.65	0.03	111.50	6.70	102.35	1.65	123.82	19.17	0.91	0.02

### Discussion experiment 3

As expected, participants’ recognition was best when the body and face showed the same expression. Although in this task participants were asked to focus on the body posture, the face still attracted substantial attention. A possible explanation is that they were uncertain about the body emotion and checked the face in search of clarification. Indeed, the congruency effect on accuracy scores seemed somewhat larger for bodies than for faces. Attention was shifted away from happy cues, whether expressed by the face or the body. In experiment 2, we observed EMG effects for facial expressions. In this experiment, participants focused on the body expressions, which may be an explanation for its lack of effect.

## General Discussion

We report three experiments investigating the recognition of emotional expressions in the face and the body. In experiment 1, faces and bodies were presented and in experiment 2 and 3, the faces and bodies were combined in emotionally congruent and incongruent naturally looking, compound stimuli. The aim of these studies was to get insight into how the emotional signals from the face and those from the body posture, independently as well as jointly trigger physiological responses in the observer. Three hypotheses were tested. First, as predicted, we observed that the recognition of facial and bodily expressions was enhanced when their presentation was paired with an emotionally congruent face or body. Second, in line with our expectations, angry and fearful face and body cues attracted more attention than happy ones, independent of the context (emotionally congruent or incongruent face or body) in which they were presented. Third, as predicted, anxious participants showed enhanced pupil dilation and corrugator response to threatening cues from the face and the body. The combination of multiple measurements provides insight into the underlying processes and shows that individual differences in anxiety, as well as contextual factors influence our reaction to the emotional expression of another person. We first summarize the results before discussing the broader implications of our research.

Facial expressions were always accurately recognized but, as shown by Experiment 2, the presence of a body posture expressing the same emotion, increased recognition rates. The inverse was also true. In fact, the greatest congruency effect was observed for happy body expressions. Isolated happy body postures were recognized correctly 76% of the time. However, when combined with a happy face, these same bodies were recognized significantly better (81% correct). We observed that participants with high STAI scores more often interpreted happy body postures as threatening when the face showed an angry expression. This is in line with an earlier study which showed that anxious individuals could not ignore angry cues from the voice when interpreting facial expressions (Koizumi et al., 2011). Being anxious thus seems to influence the way social signals are interpreted. This is consistent with the literature on negative interpretation biases related to anxiety, especially in emotionally ambiguous situations (MacLeod and Cohen, 1993; Amin et al., 1998; Miers et al., 2008).

When presented with isolated facial expressions, participants fixated longest on fear expressions and more specifically on fearful eyes (Experiment 1). In Experiment 2, when categorizing facial expressions in the context of body postures, participants attended to the face in particular when it expressed anger. In Experiment 3, when categorizing bodily expressions, they attended less to the face when the body showed a threatening expression and focused more on the body when the face expressed happiness. A general pattern across experiments was that angry and fearful faces and bodies were looked at longer than happy expressions. In other words, attention was preferentially allocated to cues indicating potential threat during social interaction (Green et al., 2003; Schrammel et al., 2009).

Participants’ pupils dilated in response to all expressions, independent of the source or the specific emotion, see Bradley et al. (2008) and Schrammel et al. (2009) for similar results. Anxiety scores predicted pupil dilation triggered by viewing angry faces (see also Kimble et al., 2010; Felmingham et al., 2011).

Participants’ faces expressed a negative emotion in response to observing angry and fearful faces and expressed a positive emotion in response to happy faces. This was not the case for body postures. Magnée et al. (2007) observed a main effect of emotion (fear > happy) and a main effect of source (body > face) on the corrugator but they did not observe an interaction. Their study did not report to what extent the corrugator differentially responded to the different body expressions and therefore, comparison with the current study is difficult. As in Magnée et al. (2007), we observed a main effect of source (body > face) in Experiment 1. It is not clear what underlies this effect, but it could be that different processes than emotional synchronization are involved, such as emotion regulation or action preparation.

We show that people process bodily expressions of emotion in a similar fashion as facial expressions and that the presence of both adds up to the total percept of the emotion. Observing emotion in others is always arousing, whether the other person expresses a positive or a negative emotion. Pupil dilation seems to reflect a general appraisal of a social counterpart in terms of potential threat or reward from an interaction. The finding that anxious participants smiled and frowned simultaneously in response to an angry face illustrates that EMG activity in an emotional paradigm reflects more than emotional synchronization and that these rapid facial expressions serve as an affiliative signal that has important functions for social interaction (Fischer and Manstead, 2008; Hareli and Hess, 2012). Simultaneous measurement of the frontalis muscle could have given us more insight, especially for better differentiating between emotional expressions. Unfortunately, we were unable to measure this muscle, as it was occluded by the eye-tracker.

We show that, when it comes to fixation patterns, emotional cues, and especially those that are threatening, attracted participants’ attention more than incongruence between the two different channels. This finding is in line with previous studies which also found longer looking times at angry expressions compared to threat-irrelevant expressions (De Bonis and Baque, 1978a; de Bonis and Freixa i Baque, 1983; Schrammel et al., 2009). Moreover, visual search studies have found that angry faces are typically detected more quickly and accurately than happy ones (de Bonis and Baque, 1978b; de Bonis et al., 1999).

The role of the amygdala, an often over-active brain area in anxious individuals (Etkin et al., 2004), in modulating this aspect of behavior, is not yet clear. For example, Adolphs et al. (2005) proposed that the fear recognition deficit in a patient with bilateral amygdala damage was caused by her inability to use information from the eye region of faces. Yet the amygdala is a complex structure with a number of nuclei that have different functions and different subcortical and cortical connections. These specific functions may explain the appearance of normal behavior or its disappearance in pathological groups. We recently found that Urbach–Wiethe disease (UWD) participants with specific damage to only the basolateral nucleus of the amygdala performed like healthy controls in recognizing face or body expressions. But when shown the incongruent face-body compounds used in Experiment 2 and 3, their facial expression recognition was significantly impaired for recognition of fearful as well as for angry faces (de Gelder et al., 2012). This result shows an intriguing similarity with the pattern we found in a study of violent offenders, a group in which deficits in the amygdala has been reported repeatedly (Anderson and Kiehl, 2012). Like the UWD patients, this group showed hyper-reactivity to the negative body expressions that were not relevant for correct task performance (Kret and de Gelder, under review). Interestingly, in the former experiment there was no difference in gaze behavior between the groups. In the current study, we did not find an overall hyper-responsiveness in highly anxious subjects but we observed a specific increase in arousal in the condition where fearful faces were paired with happy bodies while gaze behavior was unaffected. The present experiments represent an important step on using combined behavioral and physiological measures in experiments that use more complex stimuli than in the past. Further research is needed to understand how the physiological parameters used here in normal participants may or may not easily map onto the behavioral patterns.

## Conclusion

Common sense tends to hold that we read facial expressions like we read words on a page, meaning that we directly and unambiguously access the meaning word by word. However, the happy, angry, and fearful faces we see leave room for interpretation, as is clearly seen in the strong influence of the body expressions on recognition accuracy. In turn, bodily expressions are not free from contextual influences either and are recognized depending on the facial expression with which they are presented. We consistently found that participants focused more of their attention on angry and fearful versus happy cues and this counted for bodies as well as for faces. Moreover, when confronted with fear and anger, participants’ corrugator muscle became more active. These effects were most pronounced as a function of increased anxiety.

## Conflict of Interest Statement

The authors declare that the research was conducted in the absence of any commercial or financial relationships that could be construed as a potential conflict of interest.
